# Supplementary material for: Effect of Fly Maggot Protein as Dietary on Growth and Intestinal Microbial Community of Pacific White Shrimp Litopenaeus vannamei
Source: Biology (Basel). 2023 Nov 15;12(11):1433. doi: 10.3390/biology12111433 (PMC10669337; doi:10.3390/biology12111433)
Supplement: Supplementary file 1 [file biology-12-01433-s001.zip › biology-2662485-supplementary.pdf]

Table S1. Feed formulation for each group. (A) Feed composition of the CK group;(B) Feed composition of the FM group; (C) Feed composition of the FF group; (D) Feed composition of the HT group, unit: %

feed ingredients	CK
fishmeal	30
soybean meal (processed GMO soybeans)	23
peanut bran	15
yeast	5
flour	20.3
soy lecithin	1
fish oil	1
soybean oil	1
choline chloride	0.6
potassium dihydrogen phosphate	1
multivitamin	1
multimineral	1
Vc Phosphatidic Acid	0.1

(A)

feed ingredients	FM
fly maggot protein	30
soybean meal (processed GMO soybeans)	23
peanut bran	15
yeast	5
flour	20.3
soy lecithin	1
fish oil	1
soybean oil	1
choline chloride	0.6
potassium dihydrogen phosphate	1
multivitamin	1
multimineral	1
Vc Phosphatidic Acid	0.1

(B)

feed ingredients	FF
fly maggot protein	30
soybean meal (processed GMO soybeans)	23
peanut bran	15
yeast	5
flour	20.3
soy lecithin	1
fish oil	1
soybean oil	1
choline chloride	0.6
potassium dihydrogen phosphate	1
multivitamin	1
multimineral	1
Vc Phosphatidic Acid	0.1

(C)

feed ingredients	HT
fly maggot protein	30
soybean meal (processed GMO soybeans)	23
peanut bran	15
yeast	5
flour	20.3
soy lecithin	1
fish oil	1
soybean oil	1
choline chloride	0.6
potassium dihydrogen phosphate	1
multivitamin	1
multimineral	1
Vc Phosphatidic Acid	0.1

(D)

Table S2. Amino acid composition of each feed group, unit: %

Amino acid type	CK	FM	FM	HT
Asp	3.79	5.30	5.69	5.50
Thr	1.57	2.18	2.78	1.97
Ser	1.42	5.08	4.99	5.32
Glu	5.79	6.79	6.59	6.35
Gly	1.75	3.77	3.39	3.30
Ala	2.20	1.86	1.69	1.73
Cys	0.32	1.79	1.77	1.76
Val	1.85	2.69	2.66	2.32
Met	0.51	0.91	0.87	0.97
Ile	1.72	2.88	2.90	2.85
Leu	3.00	3.28	3.14	3.19
Tyr	1.36	2.57	2.61	2.58
Phe	1.58	2.60	2.51	2.70
Lys	2.70	3.87	3.79	3.76
His	1.07	1.56	1.78	1.50
Arg	2.24	3.49	3.76	3.56
Pro	1.23	4.58	4.36	4.74

Note: Tryptophan was not measured.

Table S3. Nutritional composition of groups of feed, unit: %

Nutrient composition	CK	FM	FM	HT
Crude protein	30.0	31.6	30.5	31.0
Crude Fiber	5.0	9.9	10.3	10.6
Calcium	4.0	3.9	3.9	4.1
Phosphorus	1.6	2.1	2.0	2.0
Crude Ash	16.0	10.5	10.4	10.1
Water	12.0	40.0	35.0	10.0
